# Complete Resection of a Large Mediastinal Calcifying Fibrous Tumor

**DOI:** 10.1055/s-0040-1713135

**Published:** 2020-07-12

**Authors:** Géraldine L. P. Bono, Markus Lehner, Freimut H. Schilling, Nikolai Stahr, Miriam Nowack, Philipp O. Szavay

**Affiliations:** 1 Department of Pediatric Surgery, Childrens Hospital Lucerne, Lucerne, Switzerland; 2 Department of Pediatric Hematology and Oncology, Childrens Hospital Lucerne, Lucerne, Switzerland; 3 Department of Pediatric Radiology, Childrens Hospital Lucerne, Lucerne, Switzerland; 4 Department of Pathology, UniversitätsSpital Zürich Institut für klinische Pathologie, Zurich, Switzerland

**Keywords:** calcifying fibrous tumor, pericardial resection, pediatric tumor surgery

## Abstract

Calcifying fibrous tumor (CFT) is a benign tumor entity which can present in a variety of different sites. Till date, eight cases with a mediastinal manifestation have been published in literature. Surgical removal is the treatment of choice for this often incidentally detected tumor. Surgery of thoracic CFT may be challenging due to its localization within the mediastinum. A 10-year old boy with a right-sided thoracic pectus carinatum-like deformity was referred for further evaluation, incidentally, revealing a mediastinal mass in computed tomography (CT). Laboratory results were all within normal range. Magnetic resonance imaging (MRI) showed a large tumor in the upper anterior mediastinum suggesting expansive but not infiltrative character. The tumor was displacing surrounding structures like the heart and the diaphragm. Lower venous stasis with dilation of the inferior cava vein could be demonstrated. The tumor was considered to be of benign dignity and surgical removal was indicated. Complete tumor resection could be achieved through a sternotomy approach, along with thymectomy. A partial resection of both the pericardium and diaphragm was required due to adhesion with soft tissue at those sites. The specimen's size was 320 mm × 145 mm × 100 mm, histologically confirmed as CFT. The patient showed no residual tumor at 3- and 9-month follow-up. This case is a report on a large mediastinal CFT which underwent successful complete surgical removal. Following tumor resection, prognosis is considered to be good; however, key issue is complete resection to avoid local tumor recurrence.

## Introduction

Calcifying fibrous tumor (CFT) is a rare benign collagenized lesion. It typically presents with hypocellular fibroblastic proliferation, scattered psammomatous and/or dystrophic calcifications and lymphoplasmacytic infiltration. 1 It was first described by Rosenthal and Abdul-Karim 2 in 1988. It may occur at any age and in a wide variety of sites, including many soft tissue locations, visceral organs (heart, small bowel, stomach, and adrenal gland), and all mesothelial-lined cavities. It usually presents as a painless solitary, seldom multifocal mass. Irrespective of the controversial etiology and pathogenesis of the tumor, the only therapeutic and usually curative approach is complete tumor resection. Only eight previous publications have described incidences of CFT in the mediastinum, 3 4 5 6 7 8 9 10 ranging in size from 35 to 131 mm. To our knowledge, we present the successful resection of the largest mediastinal CFT described so far.

## Case Report

**Fig. 1:**
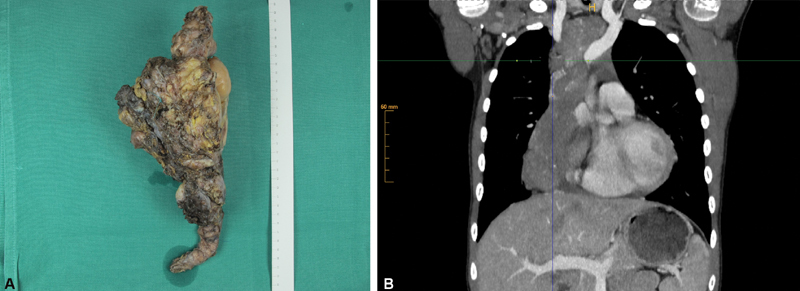
( **A** ) Macroscopic aspect of the tumor after complete surgical removal, the ruler indicating every centimeter with numbers, measuring a total length of 32 cm; ( **B** ) Preoperative computed tomography displaying the longitudinal dimension of the tumor, H indicating tumor height, showing its embedment around large thoracic vessels.

A 10-year-old boy presented with painless swelling of the right chest, diagnosed as a pectus carinatum-like chest wall deformity, slowly increasing in size over 5 years. Chest X-ray and computed tomography (CT) incidentally revealed a mass in the upper anterior mediastinum ( Fig. 1B ). The patient had previously been healthy, with an insignificant medical history. Besides an indolent swelling of his right chest, other assumed symptoms, such as chest pain, dyspnea, systolic heart murmurs, or shortness of breath, were not present in our patient.

Diagnostic assessment: further diagnostic workup included a complete physical examination, laboratory examinations, including β-human chorionic gonadotropin, and α-fetoprotein as unspecific tumor markers, to be within normal range. Sonography of the abdomen showed signs of hepatomegaly due to venous stasis caused by the mediastinal tumor but no splenomegaly or enlarged lymph nodes. Additional tumor staging imaging included a magnetic resonance imaging (MRI) of the thorax with contrast medium. It revealed a large tumor in the upper anterior mediastinum with irregular absorption of contrast medium, circumferentially surrounding the brachiocephalic trunk, with the right internal mammary artery as the main feeding vessel. The mass was extruding the heart into the left hemithorax, pushing aside the ventral thoracic wall, and lowering the right diaphragm into the left liver segments II and IV. This caused lower venous stasis. The diagnostic workup suggested an expansive but not invasive growth of the tumor. At an interdisciplinary assessment, a unanimous decision against a biopsy or any neoadjuvant therapy but for a complete surgical tumor removal was made.

**Fig. 2:**
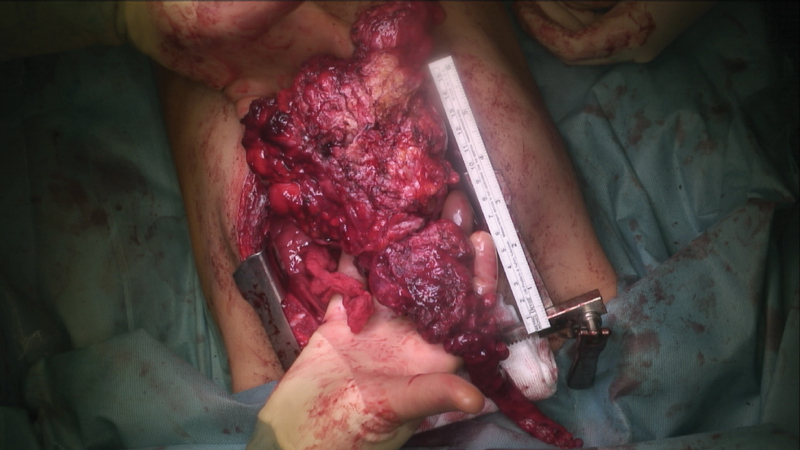
Clinical appearance of the calcifying fibrous tumor during median sternotomy.

Intraoperative course: the patient underwent a median sternotomy, resecting the mass from the brachiocephalic vein along the common carotid artery on both sides, performing a simultaneous thymectomy. An inseparable adhesion of the tumor with the right pericardium required a partial resection of the pericardium. Further inseparable adherence of the caudal aspect of the tumor with the right diaphragm also required a partial resection of the right diaphragm. This resulted in a diaphragmatic defect of 80 mm × 80 mm, which was closed later during the procedure with a Gore-Tex Patch. Adherence of the tumor with the aorta and superior cava vein above the pulmonary vessels required meticulous preservation of the large vessels, resulting in a complete extirpation of the tumor ( Fig. 2 ). Total operating time was 318 minutes.

The resected tumor showed a polylobulated, beige-yellowish surface, ranging from firm, elastic to cartilaginous hard consistency, 320 mm × 145 mm × 100 mm in size and 637 g in weight ( Fig. 1A ). Histological examination revealed a hypocellular lesion with hyalinized, collagenous stroma, lymphocellular inflammation, and psammomatous calcifications. Immunostaining and molecular pathology showed no evidence for immunoglobulin (Ig)-G4-associated diseases and no ALK (anaplastic lymphoma kinase gene)-rearrangement. The resected thymic tissue showed no abnormalities. The results coincide with the diagnosis of CFT. The boy's postoperative course was generally good, remaining 2 days in the intensive care unit and being discharged on the postoperative day 9.

Follow-up: at the 3- and 9-month follow-ups, routine blood testing, X-ray, and MRI of the thorax showed no residual tumor and no signs of tumor recurrence. The boy is currently healthy with full cardiopulmonary resources. Further follow-up with routine blood testing, chest X-ray, and MRI has planned at a s6-month interval.

## Discussion

The very rare benign CFT has been described in literature more than 180 times, 38 cases in children and adolescents from the age of 0 to 19 years. 3 4 5 6 7 8 9 10 11 Its etiology is still undetermined, although an association with IgG4-related diseases, such as Castelman's disease, has been suggested. 12 With varying location, the tumor typically presents embedded into its regional structures. Histologically with abundant dense collagenous tissue in whorled arrangements with low cellularity, identifiable by its psammomatous calcification and lymphoplasmacytic infiltrates. 1 CFT tends to originate from the internal viscera or pleura, with only eight other cases of mediastinal presentation described so far, with a mean tumor size of 82.875 mm in the long diameter (range: 35–131 mm). 3 4 5 6 7 8 9 10 To our knowledge, with a longitudinal diameter of 320 mm, the presented case is the largest mediastinal CFT described so far. A recent review of all CFT cases described until 2015, stated tumor recurrence in 10 out of 96 patients with no death owning to CFT. 11 To diagnose CFT, preferred diagnostic examination includes CT, laboratory testing, and biopsy, latest allowing the final diagnosis through histopathological examination. In this case, however, in synopsis of the conducted diagnostics, assuming an expansive but not infiltrative growth of the tumor, a benign character of the tumor was considered. Thus surgical removal of the tumor was planned. We decided to perform a median sternotomy, open surgical excision being the main treatment of bulky diseases. Due to tumor adhesion with surrounding structures, parts of the pericardium and the right diaphragm had to be resected to achieve complete resection. The patient showed no postoperative short- or long-term complications with a postoperative normal age-related cardiopulmonary function. Histological examination diagnosed the tumor as CFT, reassuring a correct treatment with complete removal.

## Conclusion

We report the largest presentation of mediastinal CFT so far, which could be surgically removed successfully. This included its adherences with the pericardium, the ventral thoracic wall, and the right diaphragm, opposing a challenging surgery by its concomitant encasement of the large vessels in the thorax. Postoperatively, the patient is healthy without any medical therapy necessary at the moment, further requiring close follow-up at a 6-month interval to rule out tumor recurrence.

## Notes

New Insights and the Importance for the Pediatric Surgeon Differential diagnosis of enlarging mass revealed incidentally by clinical or imaging examination should include calcifying fibrous tumor. At time of diagnosis, calcifying fibrous tumor (CFT) usually presents embedded in surrounding soft tissue. In our case, parts of the pericardium and of the right-sided diaphragm had to be sacrificed to achieve complete tumor resection and thus providing adequate surgical therapy for the patient.
